# Naturally Occurring Mutations in the Nonstructural Region 5B of Hepatitis C Virus (HCV) from Treatment-Naïve Korean Patients Chronically Infected with HCV Genotype 1b

**DOI:** 10.1371/journal.pone.0087773

**Published:** 2014-01-29

**Authors:** Dong-Won Kim, Seoung-Ae Lee, Hong Kim, You-Sub Won, Bum-Joon Kim

**Affiliations:** Department of Biomedical Sciences, Microbiology and Immunology, and Liver Research Institute, College of Medicine, Seoul National University, Seoul, Korea; University of Cincinnati College of Medicine, United States of America

## Abstract

The nonstructural 5B (NS5B) protein of the hepatitis C virus (HCV) with RNA-dependent RNA polymerase (RdRp) activity plays a pivotal role in viral replication. Therefore, monitoring of its naturally occurring mutations is very important for the development of antiviral therapies and vaccines. In the present study, mutations in the partial NS5B gene (492 bp) from 166 quasispecies of 15 genotype-1b (GT) treatment-naïve Korean chronic patients were determined and mutation patterns and frequencies mainly focusing on the T cell epitope regions were evaluated. The mutation frequency within the CD8+ T cell epitopes was significantly higher than those outside the CD8+ T cell epitopes. Of note, the mutation frequency within predicted CD4+ T cell epitopes, a particular mutational hotspot in Korean patients was significantly higher than it was in patients from other areas, suggesting distinctive CD4+ T cell-mediated immune pressure against HCV infection in the Korean population. The mutation frequency in the NS5B region was positively correlated with patients with carrier-stage rather than progressive liver disease (chronic hepatitis, liver cirrhosis and hepatocellular carcinoma). Furthermore, the mutation frequency in four codons (Q309, A333, V338 and Q355) known to be related to the sustained virological response (SVR) and end-of treatment response (ETR) was also significantly higher in Korean patients than in patients from other areas. In conclusion, a high degree of mutation frequency in the HCV GT-1b NS5B region, particularly in the predicted CD4+ T cell epitopes, was found in Korean patients, suggesting the presence of distinctive CD4+ T cell pressure in the Korean population. This provides a likely explanation of why relatively high levels of SVR after a combined therapy of pegylated interferon (PEG-IFN) and ribavirin (RBV) in Korean chronic patients with GT-1b infections are observed.

## Introduction

According to the WHO, 3% of the global population is infected with the hepatitis C virus (HCV), with 3–4 million people newly infected each year [1]–[4]. Most HCV infections persist, with up to 80% of all cases leading to chronic hepatitis associated with liver fibrosis, liver cirrhosis (LC) and hepatocellular carcinoma (HCC) [5]–[7]. A combinatorial treatment with pegylated interferon (PEG-IFN) and ribavirin (RBV) provides good clinical efficacy in patients infected with genotypes (GTs) 2 and 3 but is less efficacious in patients infected with the most prevalent GT-1b, thereby emphasizing the urgent need for more effective specifically targeted antiviral therapies for GT-1b [8]–[11].

The HCV RNA-dependent RNA polymerase (RdRp) is an essential enzyme that lacks proofreading activity, thus leading to a population of distinctive but closely related viral variants, termed viral quasispecies, within an infected individual [12]–[14]. Monitoring of the diversity of HCV quasispecies is important for the prediction of liver disease progression as well as HCV treatment outcomes [15]–[19]. Currently, studies regarding HCV quasispecies mainly focus on structural genomic regions; therefore, relatively limited data are available regarding nonstructural regions. Recently, variations in the nonstructural 5B (NS5B) protein, particularly in specific codons, were reported to be positively related to a sustained virological response (SVR) and end-of treatment response (ETR) of patients infected with GT-1b [15], [16].

It was also reported that the SVR rate in patients with HCV GT-1b treated with PEG-IFN plus RBV are higher in Asian patients as compared with Caucasians [10], [20]. In particular, previous studies have shown that SVR rates in Korea patients infected with GT-1b range from 56% to 62% [21], [22]. Recently, two SNPs, rs12979860 and rs8099917 of the IL28B gene, showing the strongest association with treatment response, have been reported at a high frequency in Korean patients with HCV GT-1b compared to the frequencies of other ethnic groups [23], [24]. Although prior investigations can partly explain the high SVR rates in Korean patients, other mechanisms may also contribute to this effect. In the present study, to address this issue, we investigated via quasispecies analysis the mutation frequencies and patterns in the partial NS5B from Korean patients infected with HCV GT-1b, as these are known to be related to the SVR rates,

## Methods

### Patients and HCV RNA Extraction

Serum samples were collected from a total of 73 treatment-naïve HCV-positive patients who visited Seoul National University Hospital in 2003. The clinical statuses of the HCV-positive patients were defined as carrier (C), chronic hepatitis (CH), LC or HCC. General definitions of the C and chronic liver disease types are as follows: the diagnosis of C can be made in the presence of positive anti-HCV antibodies, of a positive HCV RNA by RT-PCR, and of normal alanine aminotransferase (ALT) levels (<40 IU/L, assay dependent) in at least three tests carried out at least two months apart over a period of six months [25], [26]; CH was defined as an elevation of or fluctuation in serum ALT levels over 6 months without any evidence of any other chronic liver disease [27]; LC was diagnosed through evidence of clinically relevant portal hypertension (esophageal varices and/or ascites, splenomegaly with a platelet count of 100,000/mm^3^) [28], ultrasonographic imaging features suggestive of liver cirrhosis [29], and a histological diagnosis with one of the following features: nodular regeneration, fragmentation of the biopsy with fibrosis at the margins and a wide postnecrotic collapse with an abnormal relationship between portal tracts and central veins, and evidence of active liver-cell hyperplasia [30]. Finally, HCC in cirrhotic patients was diagnosed either through radiological criteria (focal lesion >2 cm with arterial hypervascularization according to two coincident imaging techniques) or through combined criteria (focal lesion >2 cm with arterial hypervascularization according to one imaging technique associated with AFP levels >400 ng/ml) [31]. HCV RNA was purified using the Viral Gene-Spin Viral DNA/RNA Kit (iNtRON Biotechnology Inc., Seongnam, Korea) according to the manufacturer's guideline. This work was approved by the institutional review board of Seoul National University Hospital (IRB No. C-1304-032-479). The experiment was mainly based on the viral RNA extracted from isolates; therefore, the research was done without informed consent and a waiver of informed consent was agreed upon by the IRB.

### Quantitative PCR (qPCR) and cDNA synthesis

A qPCR method was used to analyze viral RNA with an ABI7500 system (Perkin-Elmer Applied Biosystems, Warrington, UK). The primers were designed to amplify the NS2 region and the sequences were as follows: sense primer HCVF (5′-CGA CCA GTA CCA CCA TCC TT-3′) and antisense primer HCVR (5′-AGC ACC TTA CCC AGG CCT AT-3′). For the detection of HCV RNA, the SensiFAST SYBR Lo-ROX kit (Bioline, Taunton, MA, USA) was used according to the manufacturer's instructions. Absolute quantification of extracted HCV RNA relies on the accuracy with the amount of HCV RNA standard measured with a lower limit of detection of 1,350 copies/ml (500 IU/ml) on the basis of earlier research (data not shown) [32], [33]. Viral cDNA synthesis for Reverse-transcriptase (RT) PCR was done using the Maxime RT PreMix kit (iNtRON Biotechnology Inc., Seongnam, Korea) according to its own protocol.

### Nested PCR Amplification

The nested PCR method and primer pairs to amplify GT-1 to 4 are available in the literature [34]. Briefly, as an example of GT-1b, the first round of amplification was carried out using the sense primer A1b (O/S) (accession no. M62321, positions 8113–8135, 5′ - CTGACRACTAGCTGYGGTAAYAC - 3′) and the antisense primer F1b (O/A) (positions 8678–8699, 5′ - CCTGGAGAGTAACTRTGGAGTG - 3′). The first-round reaction was subjected to 30 cycles of amplification (30 s at 94°C, 30 s at 45°C and 50 s at 68°C) followed by 7 min of extension at 72°C. The second round of amplification was carried out using the sense primer B1b (I/S) (positions 8181–8205, 5′ - GCTCCRGGACTGCACSATGCTCGTG - 3′) and the antisense primer E1b (I/A) (positions 8654–8675, 5′ - AATGCGCTRAGRCCATGGAGTC - 3′) to amplify 495 bp of the GT-1b NS5B region. The second-round reaction was subjected to 30 cycles of amplification (30 s at 93°C, 30 s at 55°C and 1 min at 68°C) followed by 7 min of extension at 72°C.

### Cloning and Sequencing Analysis

The PCR products of GT-1b were cloned using the TOPO TA Cloning kit (Invitrogen Corporation, Carlsbad, CA, USA). The NS5B regions were sequenced using the M13 primer. For each subject, 10 to 12 subclones were sequenced [35], [36]. Sequencing was conducted using the Applied Biosystems model 377 DNA automatic sequencer (Perkin-Elmer Applied Biosystems, Warrington, UK). If there were sequence variations between the clones of a sample, the dominant sequence at each position was determined as the major sequence. Nucleotides were aligned and their similarities were calculated using the multiple-alignment algorithm in Megalign (DNASTAR, Windows Version 3.12e). A mutation in this study was defined as a sequence different from the consensus sequence of 20 GT-1b reference strains obtained from the LANL HCV database (http://hcv.lanl.gov) [accession numbers AB442219, AB691953, AF165047, D11168, D13558, D16435, D50485, D85516, D90208, EU256084, EU482859, FJ478453, HQ110091, HQ912958, J238799, L02836, M58335, M96362, S62220 and X61596] [37]. Because at aa 316 and 464, the two types of subclonal amino acids were conserved in each subject, both amino acids were considered to be a consensus sequence [38], [39]. For a further comparison of the analyzed sequences, 45 HCV GT-1b sequences from other countries (China: 15, Japan: 15, Switzerland: 15 and the United States: 15) were also retrieved from the LANL HCV database and relevant nucleotide positions were compared with the consensus sequence of 15 subjects.

### Phylogenetic analysis

HCV GTs were confirmed by a phylogenetic analysis based on 12 reference strains representing each of the GTs of 1–6 obtained from GenBank [accession numbers AF009606 (1a), AF064490 (5a), AY232745 (2b), D63821 (3a), D90208 (1b), DQ480515 (6a), JX961069 (2a), M58335 (1b), M67463 (1a), S62220 (1b), X61596 (1b) and Y11604 (4a)]. Phylogenetic trees were inferred using the neighbor-joining method [40]. Neighbor-joining was carried out using MEGA version 4.0.2 [41]. The resultant neighbor-joining tree and topology were evaluated by bootstrap analyses based on 1,000 re-samplings.

### Prediction of novel CD4+ T cell epitopes and determination of mutations inside and outside CD4+ or CD8+ T cell epitopes

15-mer peptides containing an association between a particular HLA class II molecule and the sequenced NS5B with binding capacity <500 mM were screened *in silico* for the presence of the relevant HLA-binding motif [42]. Mutations within the CD4+ and CD8+ T cell epitopes were defined as a sequence different from the consensus sequence within the four selected CD4+ T cell epitopes with above criteria and six known CD8+ T cell epitopes, respectively, on the basis of previous studies [37], [43]–[45]. Mutations outside the CD4+ or CD8+ T cell epitopes were counted according to the total number of mutations minus the sum of the respective epitope regions.

### Statistical analyses

The results were expressed as percentages, means ± SD, or as medians (range). The differences between the categorical variables were analyzed using Fisher's exact test or a Chi-square test. For continuous variables, the Student's t-test was used when the data showed a normal distribution, or the Mann-Whitney U test was used when the data was not normally distributed. The level of significance of each test was adjusted for multiple tests via Bonferroni correction. A p-value of <0.05 (two-tailed) was considered to be statistically significant. Statistical analysis of the data was performed using SPSS version 20 (IBM Corp. Armonk, NY, USA).

## Results

### Nested PCR-based distribution of GTs

Among 73 treatment-naïve samples which were subject to cDNA synthesis, 23 (31.5%) were amplified. The quantification cycle (Cq) values of 50 samples not amplified (33.91±1.55) were significantly lower than those of the 23 amplified samples (32.76±1.17) (p = 0.002). Similar to the serological prevalence, GT-1b (15 patients, 65.2%) and 2 (6 patients, 26.1%) were dominant in Koreans [46]. GT-3a and 4 were amplified in one subject each (4.3%) (Table S1). Clinical details of the GT-1b patients for whom the sequences were amplified are presented in Table S2.

### Phylogenetic analysis of GT-1b and its characteristics

**Figure 1:**
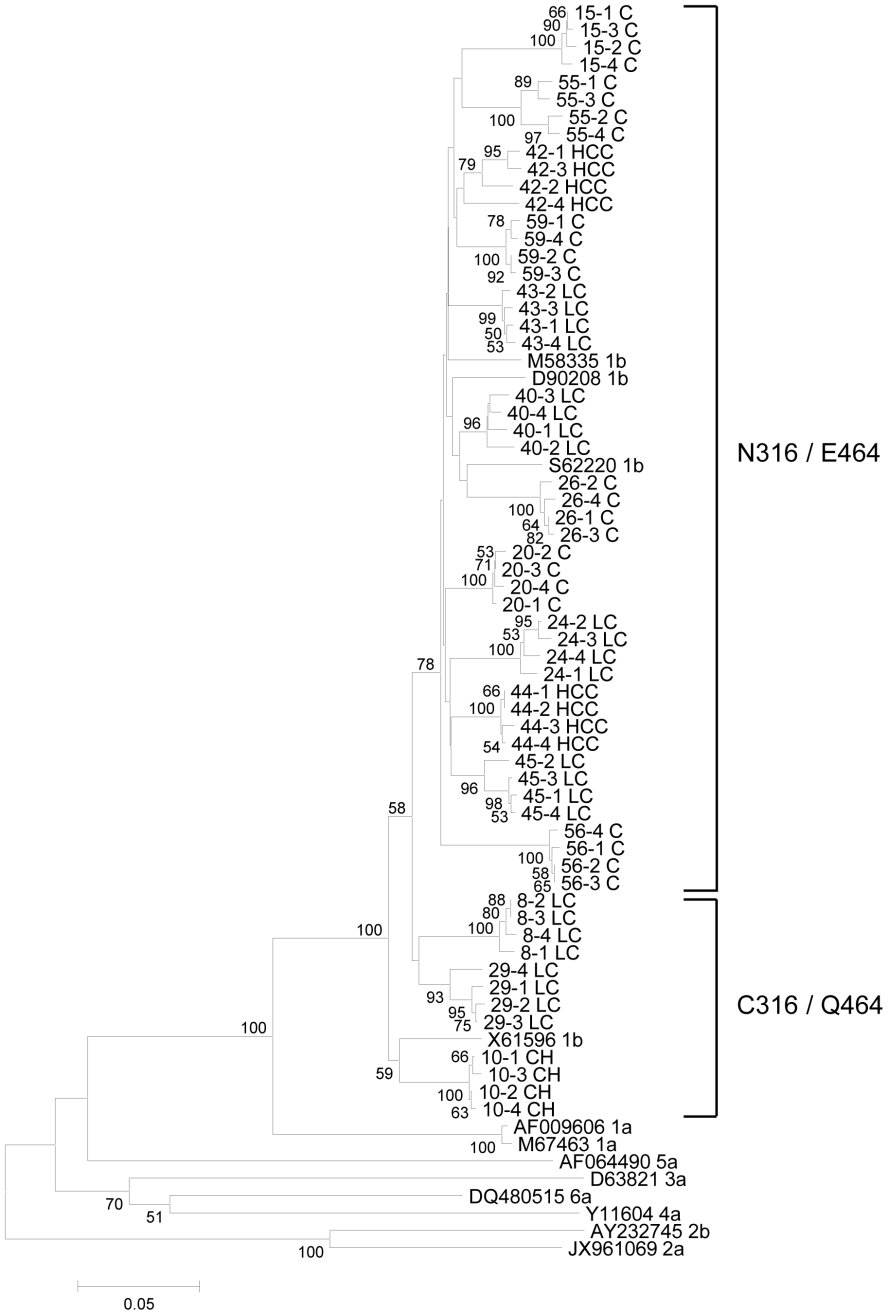
Phylogenic tree of 492bp of the GT-1b NS5B region. Genetic distances were estimated using the Kimura two-parameter matrix and the phylogenetic tree was constructed using the neighbor-joining method. The percentages indicated at the nodes represent bootstrap levels supported by 1,000 re-sampled data sets. Bootstrap values of <70% are not shown.

A phylogenetic analysis based on the 492bp GT-1b sequenced NS5B region of randomly selected subclones showed distinct sequence variation between each subject (Fig. 1). All 15 subjects belonged to GT-1b. 12 subjects with N316/E464 and three subjects with C316/Q464 were phylogenetically segregated, showing high bootstrap values (75%). The C316/Q464 group had significantly lower Cq values [C316/Q464 (31.69±0.92) vs. N316/E464 (32.28±1.05), p = 0.033] and exclusively showed advanced liver disease (CH + LC + HCC) (100%) compared to the other group (48.1%, p = 0.001) at the subclonal level (Table 1, Table S3). This finding indicates a positive correlation between viral replication and the clinical severity of liver disease. The nucleotide sequence of 166 subclones is available in the GenBank nucleotide sequence databases with the following accession numbers: KF422017-KF422027.

**Table 1:** Mutation frequency at the major codons including antiviral resistance and SVR-related codons in the sequenced NS5B region.

Amino acid variant types	No. of mutations (Total = 166)	Rate (%)	No. of patient	Mutation type^a^	Note (Reference)
Q309R	96	57.8	15	Diverse	SVR and ETR 27%, NR: 9% in Japan (15)
C316N^b^	133	80.1	12	Conserved	63.3% with C316N/Y in patients treated with IFN/RBV in Japan (15)
A333E/V	16	9.6	3	Conserved (E), Diverse (V)	SVR and ETR 23%, NR: 9% in Japan (15)
S335N	63	38.0	7	Diverse	29.6% with S335A/N in patients treated with IFN/RBV in Japan (15)
V338A	46	27.7	6	Diverse	SVR and ETR 18%, NR: 5% in Japan (15)
P353L	57	34.3	7	Diverse	17.3% with S335A/N in patients treated with IFN/RBV in Japan (15)
Q355K/R	34	20.5	3	Conserved	SVR and ETR 25%, NR: 5% in Japan (15)
E440D/G/K	40	24.1	7	Conserved (D), Diverse (G/K)	Immunosuppressant resistant mutation [58]
C451H/T/Y	46	27.7	6	Conserved (T/Y), Diverse (H)	The frequency of 2 AA are almost same in Japan (16)
E464Q^b^	33	19.9	3	Conserved	The frequency of 2 AA are almost same in Japan (16)

a. ‘Diverse’ indicates the mutation type of the coexistence with the wild type in a quasispecies distribution of a subject. Otherwise, ‘Conserved’ indicates the presence of only mutation types alone without the wild type in a quasispecies distribution of a subject.

b. C316/Q464 and N316/E464 were found in an exclusive manner. All the 33 subclones with the C316/Q464 type were found in patients with advanced liver disease, but not in Cs.

### Distribution of mutations in the sequenced NS5B region

**Figure 2:**
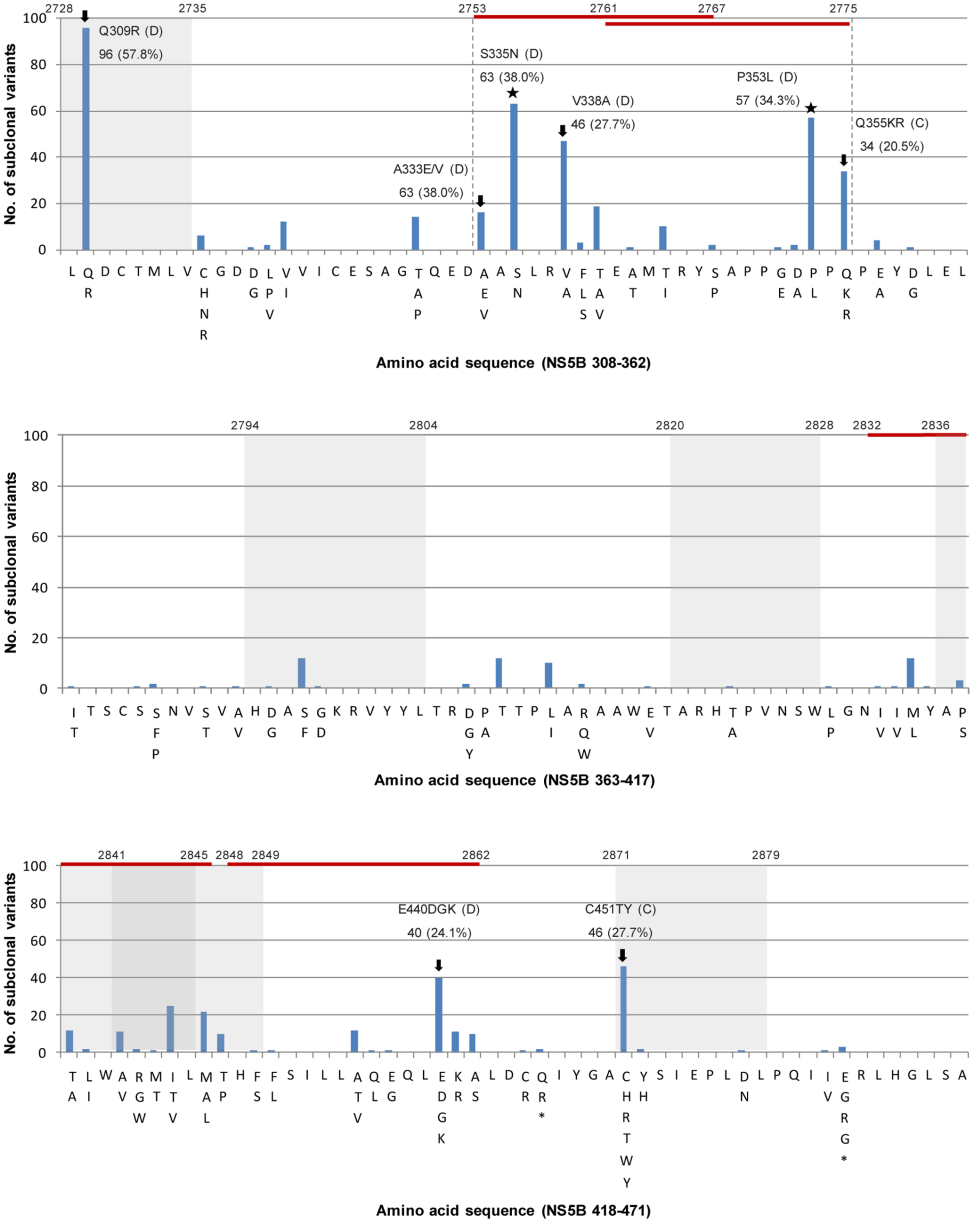
Distribution and frequencies of the amino acid mutations in the NS5B AA 308–471 regions. The blue-shaded regions are the known MHC class I restricted regions and the red-lined regions are regions expected to be Korean-specific CD4+ epitope-binding regions. The region between the dotted lines is a mutational hotspot. The arrow indicates amino acid substitution related to IFN/RBV and other agents in HCV and the asterisks denote the novel mutations found in this study. The letters C and D indicate ‘conserved’ and ‘diverse’ in the subclones, respectively.

The distribution of the mutations from the sequenced GT-1b NS5B region aa 164 is shown in Fig. 2. There were six known CD8+ T cell epitopes (Table S4) [37], [43]–[45], and the mutation frequencies inside the CD8+ T cell epitope regions (2.9%) were significantly higher than those outside the epitope regions (2.3%, p = 0.001). The mutation frequencies inside the predicted CD4+ T cell epitopes (4.8%) were significantly higher than those outside the CD4+ T cell epitope (1.4%) and were even higher than those inside the known CD8+ T cell epitopes (p<0.001) (Table S5). We designated the region including the aa 333–355 section of the CD4+ T cell epitopes as a mutational hotspot, as which an extraordinary high mutation frequency (6.7%) was observed (Fig. 2). Of note, the region was predicted to have high binding affinity for the various MHC class II HLA types prevalent in Koreans, raising the possibility that there may be distinctive MHC class II restricted immune pressure against HCV GT-1b in the mutational hotspot (Table 2).

**Table 2:** Comparison of mutation rates between the CD4+ and CD8+ T cell epitope regions.

Regions (n = 27,224)	No. of mutations/No. of codons	Mutation rate (%)	*P*-value
Outside known CD8+	425/18,758	2.3	-
Inside known CD8+	249/8,466	2.9	0.001^a^
Outside predicted CD4+	250/18,426	1.4	-
Predicted CD4+	424/8,798	4.8	<0.001^b^
Mutational hotspot_1	254/3,818	6.7	<0.001^b^

a. P-values were determined by a comparison of mutation rates of inside and outside of the CD8+ T cell epitopes.

b. P-values (<0.001) were obtained by a comparison of the mutation rates of outside of the CD4+ T cell epitopes as well as inside the CD8+ T cell epitopes.

### Comparison of synonymous (d_S_) and nonsynonymous mutations (d_N_) according to the NS5B region

The distinctive CD4+ T cell-mediated immune pressure was examined by comparing d_N_ to d_S_. The d_N_/d_S_ ratio inside the known CD8+ T cell epitopes (0.29) was slightly higher than that of the outside (0.21) region with d_N_ frequencies of 2.9% and 2.3%, respectively. The d_N_/d_S_ ratio inside the predicted CD4+ T cell epitopes (0.49) was statistically higher than that outside (0.13), with d_N_ frequencies of 4.8% and 1.4%, respectively, although the d_S_ frequency outside the predicted CD4+ T cell epitopes was higher at a statistically significant level. The odds ratio of the d_N_ inside and outside the predicted CD4+ T cell epitopes was 3.55. In the mutational hotspot, d_N_ frequencies (11.1%) were found to be higher than d_S_ frequencies (4.8%), resulting in an elevated d_N_/d_S_ ratio (1.4). This suggests there are strong MHC class II restricted immune pressures against HCV NS5B in chronic Korean patients (Table 3).

**Table 3:** Frequencies of d_N_ and d_S_ according to the NS5B region.

Regions	Total	Inside	Outside	Odds ratio
Known CD8+	No. of Codons	8,466	18,758	-
(51 AA)	d_N_ (%)	249 (2.9)	426 (2.3)	1.30^a^
	d_S_ (%)	868 (10.3)	1,992 (10.6)	0.97
	d_N_/d_S_ ratio	0.29	0.21	1.34
Known CD4+	No. of Codons	8,798	18,426	-
(53 AA)	d_N_ (%)	424 (4.8)	250 (1.4)	3.55^b^
	d_S_ (%)	863 (9.8)	1,997 (10.8)	0.91^c^
	d_N_/d_S_ ratio	0.49	0.13	3.92
Mutational hotspot	No. of Codons	3,818	23,406	-
(23 AA)	d_N_ (%)	254 (6.7)	420 (1.8)	3.71^b^
	d_S_ (%)	182 (4.8)	2,678 (11.4)	0.42^b^
	d_N_/d_S_ ratio	1.4	0.16	8.90

a. p = 0.001;

b. p<0.001;

c. p = 0.01.

### Comparisons of d_S_ and d_N_ in the NS5B region between Korean patients and patients from other countries

To examine whether there was distinctive immune pressure against HCV NS5B at the CD4+ T cell level in Koreans, we compared d_S_ and d_N_ in the NS5B region between 15 Korean patients and 60 patients from other countries (China: 15, Japan: 15, Switzerland: 15 and the United States: 15). In the Koreans subjects, we used the consensus sequences of NS5B from more than 10 subclones of patients. For the patients from other countries, we used sequences retrieved from the LANL HCV database. In the NS5B region, the d_N_/d_S_ ratio for the Korean subjects (0.23) was higher than it was for those from other countries (1.4) with statistical support (p = 0.002). The d_N_ frequency (3.1) in the known CD8+ T cell epitopes from Korean patients was higher than that for the patients from other countries (2.1), but the difference was not statistically significant (p = 0.078). However, the d_N_ frequency (4.5%) in the predicted CD4+ T cell epitope regions in the Korean patients was significantly higher than that in those from other countries (2.2%) (p<0.001). The d_N_/d_S_ ratios in the predicted CD4+ T cell epitope regions were higher in the Koreans (0.52) by nearly twofold compared to those of the patients from other areas (0.26). In particularly, the difference in the d_N_ frequency between the Koreans (6.4%) and the patients from other countries (2.3%) was more pronounced in the mutational hotspot. Collectively, these results suggest the presence of distinctive CD4+ T cell mediated immune pressure against HCV NS5B in Koreans (Table 4).

**Table 4:** Comparison of the frequencies of d_N_ and d_S_ of the NS5B regions between 15 Korean patients consensus sequence and patients from other countries.

Regions	Total	Korean (n = 15)	Other countries^a^ (n = 60)	Odds ratio
Total	No. of Codons	2,460	9,840	-
(164 AA)	d_N_ (%)	57 (2.3)	142 (1.4)	1.61^b^
	d_S_ (%)	252 (10.2)	914 (9.3)	1.10
	d_N_/dS ratio	0.23	0.16	1.46
Known CD8+	No. of Codons	765	3,060	-
(51 AA)	d_N_ (%)	24 (3.1)	63 (2.1)	1.52^c^
	dS (%)	65 (8.5)	290 (9.5)	0.90
	d_N_/dS ratio	0.37	0.22	1.70
Predicted CD4+	No. of Codons	795	3,180	-
(53 AA)	d_N_ (%)	36 (4.5)	69 (2.2)	2.12^d^
	d_S_ (%)	69 (8.7)	261 (8.2)	1.06
	d_N_/d_S_ ratio	0.52	0.26	1.97
Mutational hotspot	No. of Codons	345	1,380	-
(23 AA)	d_N_ (%)	22 (6.4)	32 (2.3)	2.75^d^
	d_S_ (%)	16 (4.6)	53 (3.8)	1.21
	d_N_/d_S_ ratio	1.38	0.60	2.28

a. China: 15, Japan: 15, Switzerland: 15 and the United States: 15.

b. p = 0.002;

c: p = 0.078;

d: p<0.001.

### Correlation between NS5B mutations and the severity of liver disease

The overall mutation frequency of the entire NS5B region in C (2.8%) was significantly higher than in the comparison group, patients with CH, those with liver cirrhosis LC and those with HCC (2.2%) (p = 0.002). The mutation frequency in known CD8+ T cell epitopes was also significantly higher in C than in the comparison group [C (3.4%) vs. CH + LC + HCC (2.6%), p = 0.05]. This tendency was also found in the predicted CD4+ T cell epitopes [C (5.7%) vs. CH + LC + HCC (4.2%), p = 0.001] and in the mutational hotspot [C (7.7%) vs. CH + LC + HCC (5.9%), p = 0.004] with an increased frequency of mutations at a statistically significant level. This shows that increases in the mutation rate in the NS5B region are negatively correlated with the progression of liver disease in chronic hepatitis C patients (Table 5).

**Table 5:** Comparison of the mutation rates in NS5B regions according to the clinical status of liver disease.

Regions (n = 27,224)	C (%)/No. of codons	CH+LC+HCC (%)/No. of codons	*P*-value
Total	320 (2.8)/11,316	354 (2.2)/15,908	0.002^a^
Known CD8+	119 (3.4)/3,519	130 (2.6)/4,947	0.05
Predicted CD4+	208 (5.7)/3,657	216 (4.2)/5,141	0.001^a^
Mutational hotspot	122 (7.7)/1,587	132 (5.9)/2,231	0.004^a^

a. Statistically significant after a Bonferroni post hoc analysis (p<0.05).

### Mutation frequency in codons related to SVR and ETR in Korean patients

Mutations at the 309, 333, 338 and 355 codons are reportedly related to SVR and ETR groups as compared to non-responders (NR) [15]. Interestingly, a very high mutation rate in four SVR-related codons was found in Korean treatment-naïve patients, with an average mutation frequency of 28.9% (192/664) in the quasispecies distributions. Of note, the average mutation frequency (31.7%) in four codons as calculated from 15 Korean patients was significantly higher than any of the other regions, including that from Japan (Table 6).

**Table 6:** Comparison of mutation rates in four SVR-related codons (309, 333, 338 and 355) in the NS5B region between 15 Korean consensus sequence and patients from other countries.

Countries (n = 15)	No. of mutations (n = 60)	Mutation rate (%)	*P*-value^a^
Korea	19	31.7	-
China	3	5	<0.001^b^
Japan	7	11.7	0.014
Switzerland	3	5	<0.001^b^
United States	1	1.7	<0.001^b^

a. P-values were determined by a comparison with the mutation rate from 15 Korean consensus sequences.

b. Statistically significant after a Bonferroni post hoc analysis (p<0.05).

A quasispecies analysis showed a total of 10 mutations, including SVR and antiviral resistance in the sequenced NS5B region. These can be divided into two distinct groups. One is the diverse (D) type, which coexists with other quasispecies members in a patient, and the other is made up of conserved (C) types which exist alone without a quasispecies counterpart in a patient (Fig. 2, Table 1). The coexistence of diverse quasispecies at a specific codon may be indirect evidence of an important target for immune pressure or/and viral fitness. Notably, the coexistence of Q and R at codon 309, located in one of the CD8+ T cell epitopes (aa 308 and 315), was found in all 15 Korean subjects via a quasispecies distribution analysis; this may be due to the distinct CD8+ T cell immune pressure against a region between aa 308 and 315 among Koreans (Table S6). In addition, there were other D type mutations: A333V, S335N, V338A, P353L, E440G/K and C451H. On the other hand, there were only three C types of mutations (C316N, Q355K/R and E464Q). Interestingly, in all the three C-type mutations, significantly different Cq values between two counterparts in the respective mutation type were found (Table S3).

## Discussion

The presence of distinct HLA types among an ethnic group could lead to distinct MHC class I or II restricted immune pressures within its population [37], [43], [44], [47]. Therefore, the frequency and patterns of escape variants against structural and nonstructural HCV proteins reflect the background HLA types among an ethnic group [48], [49]. The aim of the present study is to investigate the background mutation frequency and patterns of HCV NS5B, reportedly related to a high SVR, from treatment-naïve Korean patients chronically infected with GT-1b in an effort to explain the high SVR in Korean patients. The significant findings of this study are discussed below.

First, the entire mutation frequency in the sequenced NS5B region was positively correlated with Cs but not with patients showing disease progression (CH, LC and HCC) [C (2.8%) vs. CH + LC + HCC (2.2%), p = 0.002]. Furthermore, similar mutation frequencies were noted within both the CD4+ (p = 0.001) and CD8+ T cell epitope regions (p = 0.05) (Table 5). This suggests that the accumulation of multiple mutations in NS5B may be induced by vigorous and multi-specific immune pressure in the HCV-acute infection phase and may lead to the functional abnormality of HCV RdRp activity, resulting in the attenuation of HCV pathogenic potentials [19]. This strongly supports previous results which showed that mutations in NS5B were related to the high SVR and EVR of GT-1b chronically infected patients [15].

Second, a pronounced d_N_ frequency in the predicted CD4+ T cell epitopes in the NS5B region [Korean (4.5%) vs. those of patients from other countries (2.1%), p = 0.001], particularly in the mutational hotspot [Korean (6.4%) vs. other countries (3.1%), p = 0.01], was found in Korean patients but not in patients from other areas (Table 4). This suggests that there is distinct intrahepatic MHC class II restricted immune pressure at least against HCV NS5B among the Korean population [19]. Broadly directed virus-specific immune pressure at the CD4+ T cell level was recently reported to play a very pivotal role in spontaneous resolution at a very early phase of HCV-acute infection [50]. Furthermore, the presence of the multi-specific CD4+ T cell response against HCV can aid not only the induction of a vigorous antiviral CD8+ T cell response but also antibody production for the inhibition of the spread of the virus [51]. Particularly, because three codons (A333, V338 and Q355) out of four reported to be related to the high SVR are located in the mutational hotspot, the acquisition of mutations within this region induced by the distinctive Korean immune pressure at the CD4+ T cell level may contribute to the high SVR found in Korean patients infected with GT-1b. In fact, the prediction of the MHC class II HLA allele showed that a region of the CD4+ T cell epitope from NS5B, covering aa 333 to 347, one of two predicted epitopes comprising the mutational hotspot, has high binding affinity for most HLA DRB1 alleles prevalent in Korean populations [52]. In addition, HLA DQB1 03:01 and 03:02, prevalent at frequencies higher than 10% in Koreans, also are noted to be associated with viral clearance [53]–[55]. Our previous study also showed that there are distinct mutation patterns and a very high mutation frequency of the CD4+ T cell epitopes of the HBV preC/Core region in chronic Korean patients, strongly supporting the hypothesis of this study [56].

Third, the frequency of d_N_ within the CD8+ T cell epitope region of NS5B was significantly higher than that outside the CD8+ T cell epitope region [inside CD8+ (2.9%) vs. outside (2.3%), p = 0.001], suggesting the presence of immune pressure at the CD8+ T cell level against HCV NS5B among Korean patients, as shown in patients from other areas (Table 3) [37], [44], [47], [57]. However, pronounced differences in the mutation frequency between six regions of CD8+ T cell epitopes were found. Two of the six CD8+ T cell epitopes (308 to 315 aa and 451 to 459) with high binding affinity to two HLA allele types, HLA-A02:01 and HLA-A24:02, prevalent in Koreans, showed a higher d_N_ frequency compared to other epitopes [308–315: 96 (7.2%) and 451–459: 49 (3.3%)], suggesting the presence of distinct MHC class I restricted immune pressure in Korean patients [52]. Particularly, it is noteworthy that the extraordinary high d_N_/d_S_ ratio (2.04) found in a region of the CD8+ T cell epitope covering codons 308 to 315, was mainly due to the presence of frequent mutations in codon 309, one of four codons related to SVR rates (Table S4, Fig. 2). The mutation type, Q309R, is known to be frequently mutated in NS5B, particularly in Asian patients. However, even compared to Japanese patients, also an Asian country like Korea, the strikingly high mutation frequency of Q309R was observed in only the Korean patients [15], [16]. All of the 15 patients harbored this mutation in their quasispecies distribution and more than half (96/166, 57.8%) of all quasispecies from the 15 patients had the mutation type R309. Interestingly, the co-existence of both mutated and wild types, not exclusive of the existence of one type alone, was found in all 15 patients, suggesting the advantage of the coexistence of two variants in a patient over the exclusive existence of either type alone in an escape of host immune surveillance or viral fitness (Table S6). Therefore, the high frequency of the Q309R mutation in Korean patients may be induced by CD8+ T cell immune pressure which may in part provide a likely explanation for the high SVR rates in Koreans.

Finally, it is well known that mutations in NS5B can affect the HCV replication capacity [19]. We found a total of three types of mutations (C316N, Q355K/R and E464Q) which had a significant effect on HCV replication (Cq value: C316N and E464Q p = 0.033, Q355K/R p = 0.003) (Table S3). Interestingly, our quasispecies analysis showed that two polymorphisms in aa 316, C316 and N316, were strongly related to two polymorphisms in codon 464, Q464 and E464, respectively, in an exclusive manner (Figure 1). The type with both C316 and Q464 signatures showed a significantly higher HCV replication capacity and was more related to patients with advanced liver disease compared to the type with both the N316 and E464 signatures. The exclusive combination of the SNPs of two codons may be due to the structural constraint of NS5B. Furthermore, the coexistence of both types (C316/Q464 and N316/E464) was not found in any patients, suggesting that these two types may be from completely different resources and not a different quasispecies version induced by immune pressure from a patient. Our data showing phylogenetic segregation between the two types also supports the above hypothesis.

Our study has three potential limitations. First, the nested PCR protocol used in this study showed low sensitivity, with the amplification of only 23 samples out of 73 samples (31.5%). The strategies for the nested PCR protocol including primer sets and a PCR condition should be modified in the future study. Particularly, PCR negative amplifications were found with high frequencies in samples with lower HCV viral loads, suggesting novel nested PCR protocol to increase the degree of sensitivity should be applied in a future study. Second, the modest population size (15 patients) is relatively small to lead to a meaningful conclusion about the relationship between NS5B mutations and liver disease progression. Third, as single-genome amplification and an end-point dilution strategy were not utilized, the cloning strategy employed in this study is limited when used to represent genuine viral quasispecies in serum samples.

In conclusion, our data suggest that the distinct MHC class II restricted immune pressure against HCV NS5B in Korean patients leads to a pronounced high mutation frequency and distinct mutation patterns in HCV NS5B in Korean patients. This finding provides important insight into the high SVR and ETR rates during the treatment of GT-1b infected Korean patients.

## Supporting Information

Table S1 Distribution of amplified subjects by PCR targeting NS5B sequences.
(DOCX)

Table S2 Clinical features of 15 Korean patients in this study.
(DOCX)

Table S3 Comparison of Cq values between types at the major codons including antiviral resistance and SVR related in the sequenced NS5B region.
(DOCX)

Table S4 Comparison of amino acid sequences, dominant HLA allele, d_N_/d_S_ ratio between six regions of Known CD8+ T cell epitopes in the NS5B region.
(DOCX)

Table S5 Relationships of predicted CD4+ T cell epitopes of the NS5B with MHC class II HLA types prevalent in Korean population.
(DOCX)

Table S6 Quasispecies distribution at the codon 309 in 15 Korean patients.
(DOCX)
