# Supplementary material for: Naturally Occurring Mutations in the Nonstructural Region 5B of Hepatitis C Virus (HCV) from Treatment-Naïve Korean Patients Chronically Infected with HCV Genotype 1b
Source: PLoS One. 2014 Jan 29;9(1):e87773. doi: 10.1371/journal.pone.0087773 (PMC3906201; doi:10.1371/journal.pone.0087773)
Supplement: Table S1 — Distribution of amplified subjects by PCR targeting NS5B sequences. (DOCX) [file pone.0087773.s001.docx]

Table S1. Distribution of amplified subjects by PCR targeting NS5B sequences.

| Genotypes | No. of subjects NS5B were amplified (%) |
| --- | --- |
| 1b | 15 (65.2) |
| 2 | 6 (26.1) |
| 3a | 1 (4.3) |
| 4 | 1 (4.3) |
| Overall | 23 (31.5) |
| Not amplified | 50 (68.5) |
| Total | 73 (100) |
