# Supplementary material for: Naturally Occurring Mutations in the Nonstructural Region 5B of Hepatitis C Virus (HCV) from Treatment-Naïve Korean Patients Chronically Infected with HCV Genotype 1b
Source: PLoS One. 2014 Jan 29;9(1):e87773. doi: 10.1371/journal.pone.0087773 (PMC3906201; doi:10.1371/journal.pone.0087773)
Supplement: Table S2 — Clinical features of 15 Korean patients in this study. (DOCX) [file pone.0087773.s002.docx]

Table S2. Clinical features of 15 Korean patients in this study.

| Clinical factors | 15 patients (%) |
| --- | --- |
| Age in years, mean ± SD | 63.9 ± 9.8 |
| Male (%) | 7 (47.7) |
| Liver disease (No.), C:CH:LC:HCC | 6:1:6:2 |
| ALT (IU/L), mean ± SD | 60.6 ± 36.5 |
| Cq value, mean ± SD | 33.55 ± 1.52 |
| History of antiviral therapy | 0 |
