# Supplementary material for: Naturally Occurring Mutations in the Nonstructural Region 5B of Hepatitis C Virus (HCV) from Treatment-Naïve Korean Patients Chronically Infected with HCV Genotype 1b
Source: PLoS One. 2014 Jan 29;9(1):e87773. doi: 10.1371/journal.pone.0087773 (PMC3906201; doi:10.1371/journal.pone.0087773)
Supplement: Table S3 — Comparison of Cq values between types at the major codons including antiviral resistance and SVR related in the sequenced NS5B region. (DOCX) [file pone.0087773.s003.docx]

Table S3. Comparison of Cq values between types at the major codons including antiviral resistance and SVR related in the sequenced NS5B region.

| Amino acid variants | Cq values of WT mean ± SD (No of patients, %) | Cq values with Mutation mean ± SD (No of patients, %) | *P*-value |
| --- | --- | --- | --- |
| C316N^a^ | 31.69 ± 0.92 (3, 20.0) | 32.28 ± 1.05 (12, 80.0) | 0.033 |
| A333E/V | 33.17 ± 1.24 (3, 20.0) | 32.12 ± 0.50 (12, 80.0) | 0.184 |
| S335N | 32.8 ± 1.1 (7, 46.7) | 33.11 ± 1.37 (8, 53.3) | 0.669 |
| V338A | 32.94 ± 1.29 (6, 40.0) | 32.98 ± 1.15 (9, 60.0) | 0.953 |
| P353L | 32.27 ± 1.18 (7, 46.7) | 32.61 ± 1.20 (8, 53.3) | 0.307 |
| Q355K/R | 33.37 ± 0.92 (3, 20.0) | 31.32 ± 0.46 (12, 80.0) | 0.003 |
| E440D/G/K | 32.94 ± 1.28 (7, 46.7) | 32.99 ± 1.19 (8, 53.3) | 0.935 |
| C451H/T/Y | 32.97 ± 1.41 (6, 40.0) | 32.95 ± 0.89 (9, 60.0) | 0.978 |
| E464Q^a^ | 32.28 ± 1.05 (12, 80.0) | 31.69 ± 0.92 (3, 20.0) | 0.033 |

a. At aa 316 and 464, both amino acids were considered to be a consensus sequence (38,39). C316/Q464 and N316/E464 were found in an exclusive manner according to the phylogenetic analysis (Fig. 1).
