# Supplementary material for: Naturally Occurring Mutations in the Nonstructural Region 5B of Hepatitis C Virus (HCV) from Treatment-Naïve Korean Patients Chronically Infected with HCV Genotype 1b
Source: PLoS One. 2014 Jan 29;9(1):e87773. doi: 10.1371/journal.pone.0087773 (PMC3906201; doi:10.1371/journal.pone.0087773)
Supplement: Table S4 — Comparison of amino acid sequences, dominant HLA allele, dN/dS ratio between six regions of Known CD8+ T cell epitopes in the NS5B region. (DOCX) [file pone.0087773.s004.docx]

Table S4. Comparison of amino acid sequences, dominant HLA allele, d_N_/d_S_ ratio between six regions of Known CD8+ T cell epitopes in the NS5B region.

| Region | AA sequence | HLA allele (Reference) | HLA alleles in Koreans^a^ (Frequencies in Koreans, %) | d_N_ / d_S_ (ratio) | AA with high mutation frequency (No. of variants, %) |
| --- | --- | --- | --- | --- | --- |
| 308-315 (NS5B 2728-2735) | LQDCTMLV | A02:01 (38) | A02:01 (16.5), A02:06 (9.5) | 96 / 47 (2.04) | Q309R (96, 57.8) |
| 374-384 (NS5B 2794-2804) | HDASGKRVYYL | B38 (32) | B38:02 (0.9), A02:01 (16.5), A02:06 (9.5) | 14 / 214 (0.07) | - |
| 400-408 (NS5B 2820-2828) | ARHTPVNSW | B27 (39) | None | 1 / 102 (0.01) | - |
| 416-425 (NS5B 2836-2845) | APTLWARMIL | B07 (32) | None | 78 / 297 (0.26) | - |
| 421-429 (NS5B 2841-2849) | ARMILMTHF | B27 (39,40) | B27:05 (3.6) | 72 / 191 (0.38) | - |
| 451-459 (NS5B 2871-2879) | CYSIEPLDL | A24:02 (52) | A24:02 (22.9) | 49 / 94 (0.52) | C451H/R/T/W (46, 27.7) |

a. Binding capacity < 500mM were selected.
