# Supplementary material for: Naturally Occurring Mutations in the Nonstructural Region 5B of Hepatitis C Virus (HCV) from Treatment-Naïve Korean Patients Chronically Infected with HCV Genotype 1b
Source: PLoS One. 2014 Jan 29;9(1):e87773. doi: 10.1371/journal.pone.0087773 (PMC3906201; doi:10.1371/journal.pone.0087773)
Supplement: Table S5 — Relationships of predicted CD4+ T cell epitopes of the NS5B with MHC class II HLA types prevalent in Korean population. (DOCX) [file pone.0087773.s005.docx]

Table S5. Relationships of predicted CD4+ T cell epitopes of the NS5B with MHC class II HLA types prevalent in Korean population.

| Region | AA sequence | HLA alleles^a^ [%, binding level (mM)] |
| --- | --- | --- |
| 333-347 (NS5B 2753-2767) | AASLRVFTEAMTRYS | DRB1*15:01 (8.0%, 82.9), DRB1*09:01 (10.4%, 110.5), DRB1*04:05 (8.5%, 134,6), DRB1*01:01 (7.4%, 58.9), DRB1*07:01 (7.3%, 101.6), DRB1*11:01 (3.2%, 89.5) |
| 341-355 (NS5B 2761-2775) | EAMTRYSAPPGDPPQ | DRB1*09:01 (10.4%, 331.4), DRB1*15:01 (8.0%, 339.4) |
| 412-426 (NS5B 2832-2846) | IIMYAPTLWARMILM | DRB1*09:01 (10.4%, 68.4), DRB1*15:01 (8.0%, 37.9), DRB1*01:01 (7.4%, 32.1) |
| 428-442 (NS5B 2848-2862) | HFFSILLAQEQLEKA | DRB1*09:01 (10.4%, 65.8), DRB1*04:05 (8.5%, 16.8), DRB1*11:01 (3.2%, 133.1), DRB1*03:01 (2.2%, 59.9) |

a. Binding capacity < 500mM were selected.
