# Supplementary material for: Naturally Occurring Mutations in the Nonstructural Region 5B of Hepatitis C Virus (HCV) from Treatment-Naïve Korean Patients Chronically Infected with HCV Genotype 1b
Source: PLoS One. 2014 Jan 29;9(1):e87773. doi: 10.1371/journal.pone.0087773 (PMC3906201; doi:10.1371/journal.pone.0087773)
Supplement: Table S6 — Quasispecies distribution at the codon 309 in 15 Korean patients. (DOCX) [file pone.0087773.s006.docx]

Table S6. Quasispecies distribution at the codon 309 in 15 Korean patients.

| Subjects | Subclones with Q309 | Subclones with Q309 | Q309 / R309 Ratio | Sum |
| --- | --- | --- | --- | --- |
| 8 (LC) | 4 | 8 | 0.50 | 12 |
| 10 (CH) | 4 | 6 | 0.67 | 10 |
| 15 (C ) | 6 | 6 | 1.00 | 12 |
| 20 (C ) | 5 | 7 | 0.71 | 12 |
| 24 (LC) | 2 | 9 | 0.22 | 11 |
| 26 (C ) | 5 | 6 | 0.83 | 11 |
| 29 (LC) | 5 | 6 | 0.83 | 11 |
| 40 (LC) | 5 | 5 | 1.00 | 10 |
| 42 (HCC) | 3 | 9 | 0.33 | 12 |
| 43 (LC) | 4 | 7 | 0.57 | 11 |
| 44 (HCC) | 5 | 5 | 1.00 | 10 |
| 45 (LC) | 6 | 4 | 1.50 | 10 |
| 55 (C ) | 3 | 9 | 0.33 | 12 |
| 56 (C ) | 6 | 4 | 1.50 | 10 |
| 59 (C ) | 7 | 5 | 1.40 | 12 |
| Total | 70 | 96 | 0.73 | 166 |
